# MIRACLE_2_ and SCAI grade identify patients for early wakening after out-of-hospital cardiac arrest: a post hoc analysis of the THAW trial

**DOI:** 10.1186/s13054-022-04246-z

**Published:** 2023-01-06

**Authors:** Rupert Simpson, Grigoris V. Karamasis, John Davies, Nilesh Pareek, Thomas R. Keeble, Study Group Collaborating Authors, Maria Maccaroni, Max Damian, Noel Watson

**Affiliations:** 1 Essex Cardiothoracic Centre, MSE Trust, Basildon, SS16 5NL Essex UK; 2 MTRC, Anglia Ruskin School of Medicine, Chelmsford, Essex UK; 3 Second Department of Cardiology, Attikon University Hospital, National and Kapodistrian University of Athens Medical School, Athens, Greece; 4 King’s College Hospital NHS Foundation Trust, London, UK; 5 School of Cardiovascular and Metabolic Medicine and Sciences, British Heart Failure Centre of Excellence, King’s College London, London, SE5 9NU UK

Out-of-hospital cardiac arrest (OHCA) is a major unmet clinical need, with the majority of patients dying due to neurological injury [1]. Traditionally, patients were sedated and ventilated for 36 h to facilitate therapeutic hypothermia (TH) followed by neurological prognostication 72 h after admission [2]. The TTM2 trial showed no benefit for TH over normothermia for all-cause mortality at 6 months [3], prompting limitation in TH provision and prioritization of fever avoidance. This presents opportunity for earlier waking attempts on the Intensive Care Unit (ICU) but, selecting patients for early waking remains challenging with limited evidence.

We conducted a post hoc analysis of the Therapeutic Hypothermia and eArly Waking (THAW) trial [4]. This study was designed to assess safety and feasibility of discontinuing sedation and paralysing medication for early evaluation of neurological status and extubation in OHCA survivors. At 11 h suitability for early waking was assessed, based on cardiovascular (stable haemodynamics, single inotropic support), respiratory (blood gas exchange normal, Fi02 < 0.5 and PEEP 5–8 cmH_2_0), neurological (pupils equal, reactive, withdrawing to painful stimuli and no evidence of seizure activity) and metabolic (urine output > 0.5 ml/kg, normal sodium and potassium) status [4]. If suitable, neuromuscular blocking agents were discontinued, sedation weaned and anti-shivering regime commenced prior to attempted extubation.

Practical risk scoring can aid decision-making in OHCA [5] and, in our analysis, we applied the MIRACLE_2_ score on hospital admission and Society of Cardiovascular Angiography and Interventions (SCAI) shock grade on ICU admission. The primary endpoint was early waking (< 36 h from ICU admission).

Fifty adult patients met the inclusion criteria of OHCA with ROSC, 4 died < 12 h, leaving a total of 46 patients assessed for early waking at 12 h, with 23 patients suitable, and 23 not suitable. Median age was 65.8 ± 11.5 years and 82% (*n* = 41) were male. Median time to ROSC was 29 ± 24.1 min, 88% (*n* = 44) presented with a shockable rhythm and 68% (*n* = 34) received bystander CPR. We identified patients as low (0–3) and high risk (≥ 4) of poor neurological outcomes by MIRACLE_2_ score, dividing the cohort into 24 low and 22 high risk patients (Fig. 1).

**Fig. 1:**
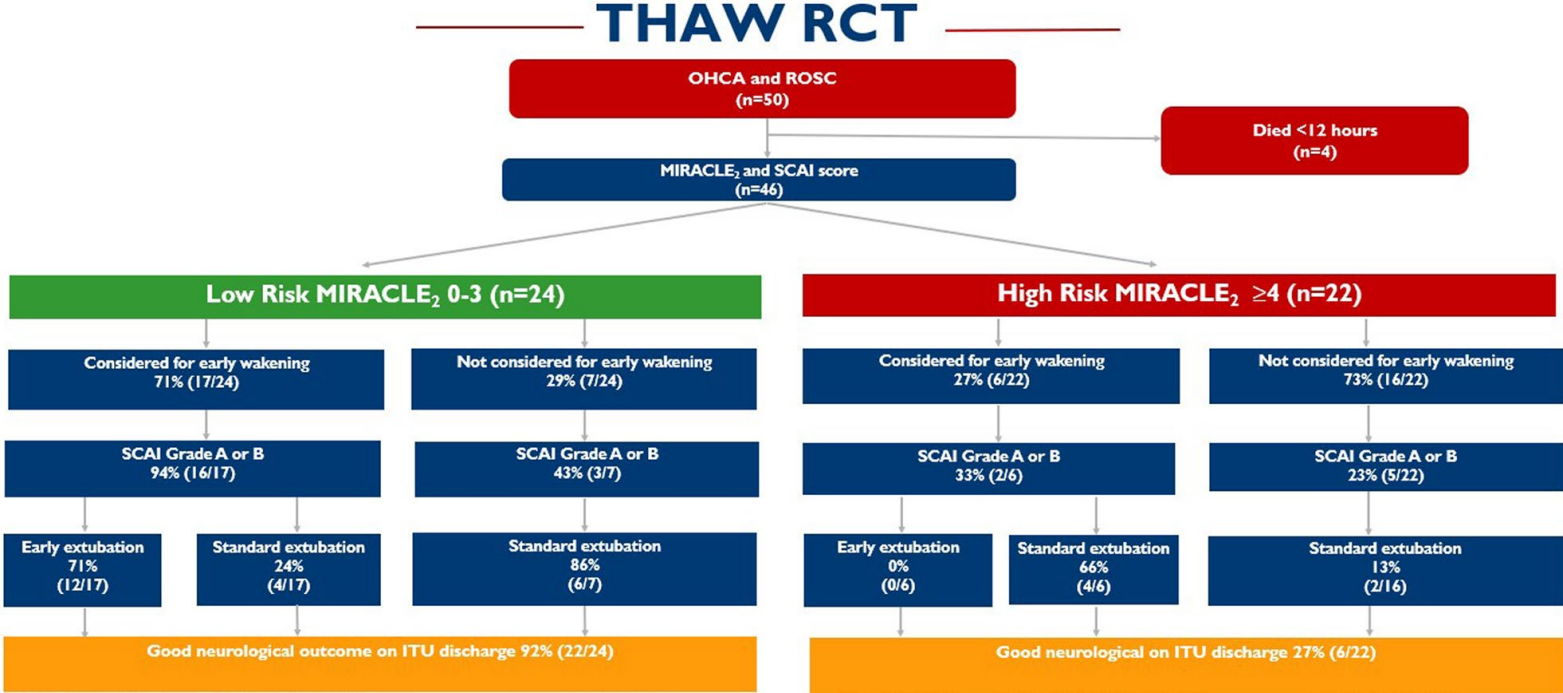
The THAW cohort with application of the MIRACLE_2_ score into low (0–3) and high-risk (≥ 4) groups

Comparing low to high-risk MIRACLE_2_ patients, there was higher suitability for early wakening [17/24 (71%) vs. 6/22 (27%)] and with 94% of low-risk patients considered for early wakening being SCAI grade A/B. Of these patients, the primary endpoint of early wakening occurred in 71% of the low-risk (12/17) and in 0% (0/6) of the high-risk groups. Of the 12 patients successfully extubated early, mean time to extubation was 21.4 ± 8.6 h, none required re-intubation and 5 patients were discharged from ICU < 48 h after admission. Good neurological outcome at ICU discharge with successful extubation occurred in 22/24 (92%) low-risk patients compared to 6/22 (27%) at high risk.

Our study indicates OHCA survivors with a low MIRACLE_2_ score (0–3) and SCAI shock grade A/B, might be amenable for early waking. This could enable shorter ventilation times, allow earlier neurology assessment and shorten ICU stay. Limitations include a small sample size and TH use, which is not necessarily applicable post TTM-2. The findings are hypothesis generating and future studies might incorporate risk stratification tools to evaluate their potential role of early extubation on ICU.

## Data Availability

Original materials from the THAW study are kept filed at the Essex Cardiothoracic Centre as per the original ethics agreement.
